# Patients and informal caregivers in the lead: a qualitative study on the experiences of patients, informal caregivers, and healthcare professionals with involvement in treatment, e-health and self-management programs

**DOI:** 10.1186/s12913-024-11156-z

**Published:** 2024-06-10

**Authors:** Matthijs H. Bosveld, Anne G.M. Smits, Helena J.M.M. Mertens, Michel J.J.M. van Zandvoort, Walther N.K.A. van Mook, Marloes A. van Bokhoven

**Affiliations:** 1 Care and Public Health Research Institute (CAPHRI; department of Family Medicine), Maastricht University, Maastricht, The Netherlands; 2 Maastricht University Medical Centre+ (board of directors), Maastricht, The Netherlands; 3 Maastricht University Medical Centre+ (Academy), Maastricht, The Netherlands; 4 Maastricht University Medical Centre+ (department of Intensive Care), School of Health Professions Education, Maastricht University, Maastricht, The Netherlands; 5 School of Health Professions Education (SHE), Maastricht University, Maastricht, The Netherlands

**Keywords:** Self-management, Self-management / methods*, Patient participation, Shared decision making, Integrated care, Informal caregivers

## Abstract

**Background:**

A significant proportion of patients and informal caregivers favor an active role in decisions concerning their health. Simultaneously, governments aim to shift treatment from a professional care setting to a community setting, in light of an ageing population, a decreasing number of health workers and allocation of scarce resources. This transition of care solicits patients’ and informal caregivers’ ability to self-manage. Therefore, the Maastricht University Medical Centre + has established the Academy for Patients and Informal caregivers. The aim is to proactively and professionally support patients and their informal caregivers to enhance their self-management. For that, the Academy offers activities in three categories: (1) instruction of nursing techniques, (2) training of e-health competencies and (3) the provision of self-management programs. Both patients with an episodic care need, as well as patients and informal caregivers with chronic illness, are eligible to participate in the Academy’s activities. However, little is known about the experience of these interventions from the perspective of patients, informal caregivers and healthcare professionals.

**Methods:**

We conducted semi-structured interviews with 15 patients, 8 informal caregivers and 19 health care professionals who either participated in, referred to or received patients from the Academy. Topics revolved around self-management and the Quadruple aim, covering topics such as patient experiences, healthcare costs, health and well-being of the population and improving work life for health professionals. Data were analyzed using thematic analysis.

**Results:**

Patients and caregivers experienced an increase in the ability to manage health needs independently, leading to increased mental well-being and self-efficacy. They felt recognized as partners in care, although managing illness needs came with its own burdens. Health care professionals indicated that they felt assured of the quality, uniformity and availability of activities due to its central organization, with instruction nurses finding greater meaning in their work. On the level of health care systems, participants in this study mentioned a decrease in use of formal healthcare, whilst enabling a more equitable division of care.

**Conclusion:**

Stakeholders’ experiences with the Academy for Patients and Informal caregivers indicate that participation contributes to development of self-management, whilst also improving working conditions, reducing the appeal to formal care and advancing equity in healthcare. The burden for patients and informal caregivers is to be considered in future developments.

**Supplementary Information:**

The online version contains supplementary material available at 10.1186/s12913-024-11156-z.

## Introduction

The nature of disease has changed tremendously over the past century. Chronic illnesses are increasingly prevalent and cause the highest burden of disease worldwide [1]. Simultaneously, an increasing shortage of health workers exists worldwide. In order to attain effective coverage of health services, the World Health Organization (WHO) reported a global shortage of 7.2 million health workers in 2013. This number is projected to grow with an additional 18 million health workers by 2030 [2]. In order to guarantee continuity of care and to optimize allocation of scarce resources, including the use of health work force, governments aim to shift treatment from a professional care setting to an informal care setting [3].

Concurrently, active and informed participation of patients in their own health care is increasingly implemented in daily practice, fueled by their own desire and ethical imperative [4, 5]. Patients’ participation in care is associated with favorable health outcomes, such as increased compliance, enhanced quality of life, and the delivery of more appropriate and cost-effective treatments [6, 7]. In more recent years, the participation of families, partners, other informal caregivers and representatives in one’s health care has been increasingly recognized as important [8, 9].

These transitions of care solicit patients’ and informal caregivers’ ability to self-manage. It can be seen in the light of a paradigm shift in health-related thinking, in which the current paradigm emphasizes the ability to adapt and self-manage [10, 11]. Self-management can be defined as a dynamic, interactive and daily process in which individuals engage to manage chronic illness [12]. It refers to the individual’s ability, together with family, community and health care professionals, to manage symptoms, treatment, physical- and psychosocial consequences, and lifestyle changes inherent with a chronic condition. Schulman-Green et al. identified three categories of self-management processes: focusing on illness needs, activating resources and living with a chronic illness [13, 14].

In order to optimize patients’ and informal caregivers’ self-management, self-management support can be deployed. In self-management support, professionals apply a patient-centered collaborative approach to promote patient activation, education and empowerment [15]. Self-management support interventions have proven to be useful and beneficial when it comes to health-related quality of life, self-efficacy, disease specific self-care behavior and cost effectiveness [16, 17]. However, many self-management support programs specifically target a single disease, focus on a single process of self-management and omit the experiences of informal caregivers. Additionally, self-management support interventions appear to be difficult to implement in practice. This is in part due to the fact that self-management support benefits from catering to individual needs, consuming scarce time and resources from the individual professional in practice [18].

To overcome this, the Maastricht University Medical Centre + established the Academy for Patients and Informal caregivers (API) in 2018, forming a comprehensive, centralized infrastructure within the organization [19]. The API proactively and professionally instructs patients and their informal caregivers to enhance their self-management, as a means to positively influence quality of care, to decrease usage of care and to support patients’ transitions to their home environments. Currently, the API employs three main categories of activities, resonating the three categories of self-management processes by Schulman-Green et al.: (1) centralized, professional instruction of nursing techniques by experienced and certified nurses, focusing on illness needs; (2) training of necessary e-health competencies to increase compliance to supportive applications and augment self-monitoring, in relation to the category of activating resources and (3) provision of comprehensive educational programs that aid in self-managing chronic illness to enhance quality of living with a chronic illness for both patients and informal caregivers [13]. The API intends to provide a palette of activities (Table 1) to optimally support patients’ and informal caregivers’ self-management.

This study qualitatively explored the experiences with the API as a form of self-management support from the perspectives of participating patients, their informal caregivers and health care professionals, in and outside of the hospital.

**Table 1:** Overview of the activities of the Academy for Patients and Informal caregivers

**Category 1: instructions of nursing techniques**
*Simple nursing techniques*
Injecting (subcutaneous and intramuscular)
Putting on compression stockings
Urinary catheter care (self-catheterization, change of day-night bag, nephrostomy catheter care)
Wound care
Drain care
Administration of ocular medication
*Complex nursing techniques*
Administration of intravenous antibiotics
Administration of enteral nutrition through a nasogastric tube
Stoma care (enteral and urinary)
**Category 2: coaching sessions for e-health competences**
Introduction to Sanacoach *SanaCoach includes several e-health applications that enable remote counselling of patients with chronic conditions, such as heart failure, chronic obstructive pulmonary disease and/or inflammatory bowel disease.*
**Category 3: educational programs for living with a chronic illness**
PPEP4ALL *PPEP4ALL is a validated self-management program aimed at improving the quality of life of patients with chronic disease and their informal caregivers* [20].
ZMILE *ZMILE is a validated self-management program aimed at improving quality of life and compliance in people with epilepsy* [21].

PPEP4ALL = Patient and Partner Education Program for All Chronic illnesses

ZMILE = Dutch abbreviation for SelfManagement Intervention in epiLEpsy

## Methods

### Study design

We took a phenomenological approach for this cross-sectional, explorative interview study. Individual, semi-structured interviews were conducted with patients, informal caregivers and professionals. Standards for reporting qualitative research (SRQR) [22] and Consolidated criteria for reporting qualitative research (COREQ) [23] were used as guides in reporting.

### Setting, participants and sampling

Both patients and their informal caregivers with an episodic care need as well as patients and their informal caregivers with chronic illness are eligible to participate in the activities of the Academy (Table 1). Patients and informal caregivers who had participated in one of the activities of the API, as well as healthcare professionals working with the API, were able to partake in this study. From the hospital setting both instruction nurses^1^ working at the API as well as referring and receiving professionals were purposively sampled. Referring professionals could both be consultants, as well as nurse(s) (practitioners), nurse team managers and care coordinators. Receiving professionals are nurses working in home care. All professionals were invited through an e-mail that included written information on the study and an informed consent form.

Additionally, instruction nurses at the API conveniently recruited patients and informal caregivers. The API nurses informed patients and informal caregivers on the study orally and asked to share contact details when interested. The principal investigator (MB) subsequently informed potential participants in more detail over the telephone. When patients or informal caregivers expressed interest to partake, an information letter and an informed consent form were provided and an interview was scheduled. No other prior relation was established with participants.

### Data collection

After obtaining written informed consent, interviews were held either in person at the hospital, in a room booked for the interview, or digitally (Microsoft Teams) using a semi-structured interview guide (see Additional files 1 and 2). The interview guide pivoted around two empirical concepts. One the one hand, the categories of self-management processes as described by Schulman-Green et al., were used to deepen experiences with self-management when brought up by the participants [13]. On the other hand, the aspects of the Quadruple Aim were explored. The Quadruple Aim is a compass to optimize health system performance through simultaneously improving patient experiences, reducing costs, improving health and improving work life for health professionals [24]. The interview guide was piloted with one patient and one informal caregiver to optimize its understandability prior to the start of data collection. The interviews were conducted by MB and AS. MB is experienced in qualitative research and moderating focus group interviews, whereas AS had limited experience with qualitative research. She was guided throughout this process by MB and MvB. Each interview was pre-discussed with MB. Up until the fourth interview, the interviews were transcribed and formulation of questions and depth of answers and subsequent follow-up questions were discussed with MB and MvB, immediately after each interview. All interviews executed by AS were deemed of sufficient quality by MB and MvB and therefore included in the analysis. Only the interviewer and the participants were present, with one exception where a spouse provided technical assistance to a patient during a digital interview. No repeat interviews were carried out. In total, there were 18 months between the first and the last interview.

All interviews were audio-recorded digitally, transcribed verbatim by MB and AS and pseudonymized (see Tables 2 and 3; and Table 4 for coding). After transcription, the audio-recordings were deleted for privacy reasons. Data collection was continued until both the principal investigator (MB) and co-researchers (AS, HvdB and MvB) agreed upon data sufficiency.

### Data analysis

A member check, consisting of a brief summary of the interview, was performed for each participant. The data were coded inductively following the principles and steps of thematic analysis [25]. The data were independently, separately coded by MB and AS for the first 33 interviews, and any differences in coding were regularly discussed. For the last 9 interviews MB initially coded the interviews, after which HvdB checked the (additional) coding. This iterative constant comparison process led to continuous additions and optimalisation of the coding tree until all interviews were analyzed. Any persisting discrepancies in coding were discussed with MvB, until consensus was reached. NVivo Pro 12.6.1.970 was used to organize and code the data [26]. An overview of the coding tree can be found as a supplement (Additional file 3).

### Reflexivity

The researchers acknowledge the fact that preconceptions, such as previous personal and professional experiences and pre-study beliefs are of influence on and inherent to the process of qualitative research [27]. To optimally tackle this, the research team consists of a members with complementary perspectives, namely clinicians, policy makers and educationalists. The participating clinicians have clinical experience both in and outside the hospital.

MB is a medical doctor and PhD student who has worked in a hospital setting and holds a master degree in healthcare policy, innovation and management. AS is also a medical doctor and participated in the research as part of her master thesis. HM is the chair of the board of directors of the Maastricht University Medical Centre + and has a background as a gynecologist. She conducted PhD research into gynecological oncology. MvZ is the director of the Maastricht University Medical Centre Academy with experience in different roles such as Human Resources Advisor and manager focusing on nursing education. WvM is an internist/intensivist at Maastricht University Medical Centre + and full professor in medical education research focusing on professional development. He holds the position of postgraduate dean. MvB is a family physician and full professor at Maastricht University, focusing on interprofessional education and collaboration in primary health care.

## Results

### Participants

In total 15 patients, 8 informal caregivers and 19 health care professionals were interviewed. Member checks were sent after each interview leading to one alteration. One participant altered the interpreted ‘cognitive capabilities’ to ‘ability and willingness to learn’. Non-participation for healthcare professionals was mostly due to time restrictions. Patients and informal caregivers declined participation mostly due to energy restrictions and/or overburdening. The duration of the interviews varied between 15 and 60 minutes.

Our sample contained patients and informal caregivers who participated in activities from the three different categories provided by the Academy. The majority of the patients and informal caregivers were between 45 and 64 years old and most of them obtained a bachelor’s degree or higher (ISCED levels 6, 7 and 8). Most informal caregivers had a spousal relation to the patient. Most participating professionals were female and worked in the hospital setting, with most of them having a background as a nurse or nurse practitioner. Professionals with experience in an extramural setting were also part of our sample.

**Table 2:** Demographics of participating patients

*N*	ID	Sex	Age	Level of education	Marital status	Paid labor	Activity (category)	Professional help after instruction?
1	P1	M	45–64	Master’s or equivalent level	Married	Yes	Injecting (1)	No
2	P2	M	45–64	Upper secondary education	Married	Yes	Compression stockings (1)	No
3	P3	M	> 65	Bachelor’s or equivalent level	Married	No	Compression stockings (1)	No
4	P4	M	45–64	Bachelor’s or equivalent level	Single	No	Compression stockings (1)	No
5	P5	F	45–64	Upper secondary education	Married	No	Injecting (1)	No
6	P6	F	45–64	Upper secondary education	Married	Yes	Injecting (1)	No
7	P7	F	45–64	Bachelor’s or equivalent level	Living together	Yes	Injecting (1)	No
8	P8	M	> 65	Upper secondary education	Widow(er)	No	Compression stockings (1)	No
9	P9	M	45–64	Upper secondary education	Married	No	Tube feeding (1)	No
10	P10	F	> 65	Upper secondary education	Widow(er)	No	Administration eye medication (1)	No
11	P11	M	45–65	Bachelor’s or equivalent level	Married	No	Administration eye medication (1)	No
12	P12	M	> 65	Bachelor’s or equivalent level	Married	Yes	E-health (2)	No
13	P13	M	> 65	Upper secondary education	Single	No	E-health (2)	No
14	P14	F	45–64	Bachelor’s or equivalent level	Married	Yes	Self-management program (3)	No
15	P15	F	> 65	Bachelor’s or equivalent level	Married	No	E-health (2)	No

**Table 3:** Demographics of participating informal caregivers

*N*	ID	Sex	Age	Level of education	Relation to patient	Paid labor	Activity (category)	Professional help after instruction?
1	IC1	F	45–64	Bachelor’s or equivalent level	Spouse	Yes	Compression stockings (1)	No
2	IC2	F	45–64	Upper secondary education	Spouse	No	Compression stockings (1)	No
3	IC3	M	45–64	Bachelor’s or equivalent level	Spouse	Yes	Injecting (1)	No
4	IC4	M	45–64	Bachelor’s or equivalent level	Spouse	Yes	Injecting (1)	No
5	IC5	F	45–64	Upper secondary education	Child	No	Compression stockings (1)	No
6	IC6	M	45–64	Master’s or equivalent level	Spouse	Yes	Self-management program (3)	No
7	IC7	F	45–64	Doctoral or equivalent level	Child	Yes	E-health (2)	No
8	IC8	M	> 65	Bachelor’s or equivalent level	Spouse	No	E-health (2)	No

**Table 4:** Demographics of participating healthcare professionals

*N*	ID	Sex	Age	Occupation	Experience with current occupation in years	Setting
1	IN1	F	45–64	Instruction nurse	1,5	Hospital
2	IN2	F	25–44	Instruction nurse	1	Hospital
3	IN3	F	45–64	Instruction nurse	1	Hospital
4	IN4	F	45–64	Instruction nurse	1,5	Hospital
5	R1	F	45–64	Care coordinator	7	Hospital
6	R2	M	45–64	Nurse team manager	4	Hospital
7	R3	F	45–64	Nurse practitioner	2	Hospital
8	R4	F	45–64	Nurse team manager	15	Hospital
9	R5	M	45–65	Consultant	10	Hospital
10	R6	F	45–64	Nurse team manager	6	Hospital
11	R7	M	25–44	Nurse team manager	3	Hospital
12	R8	F	25–44	Nurse team manager	6	Hospital
13	R9	F	25–44	Nurse practitioner	2	Hospital
14	R10	F	25–44	Nurse	2	Hospital
15	R11	F	25–44	Care coordinator	2	Home care
16	TN1	F	45–64	Transfer nurse	11	Hospital
17	TN2	F	45–64	Transfer nurse	15	Hospital
18	H1	F	45–64	Home care nurse	27	Home care
19	H2	F	25–44	Home care nurse	4	Home care

### Themes

The analysis of the interviews yielded themes on three different levels: the level of (1) patients and informal caregivers, (2) health care professionals and (3) health care systems. In the following sections, we discuss different themes for each level, displaying perspectives of all participants, substantiated with illustrative quotes. An overview of themes and subsequent thematic statements can be found in Table 5 [28].

**Table 5:** Overview of themes and thematic statements organized per level

**Level of patients and informal caregivers**
Managing illness needs
Knowledge (1) *Instructions of nursing techniques and coaching sessions for e-health competences appeared to be beneficial for patients’ and informal caregivers’ knowledge on their conditions.*
Skills (2) *Instructions of nursing techniques aided in the execution of health tasks by patients and informal caregivers.*
Monitoring health status (3) *Coaching sessions for e-health applications enabled the ability to monitor the health status for patients and informal caregivers.*
Mental well-being (4) *Instructions of nursing techniques and coaching sessions for e-health applications lead to a perceived increase of autonomy, a reduction in perceived invasion of privacy and the possibility for the informal caregiver to participate in the disease trajectory for patients and informal caregivers, with the caveat of burdening each other too much.*
Perceived advantages of self-management
Self-efficacy (5) *Activities from the three different categories lead to perceived increased self-efficacy for patients and informal caregivers.*
Peer-to-peer contact (6) *Educational programs for living with a chronic illness facilitated peer-to-peer contact for patients and informal caregivers.*
Acknowledgement (7) *Activities from the three different categories lead to the feeling of being acknowledged as a partner in care for patients and informal caregivers.*
Perceived disadvantages of self-management
Burden and complexity (8) *Instructions of nursing techniques possibly leads to an increased burden on informal caregivers, expecting that the willingness to independently execute a health task decreases when asked to execute a more complex skill.*
**Level of health care professionals**
Perceived workload (9) *Professionals working in the hospital ward experienced a reduction in workload as a result of the execution of the instruction of nursing techniques by (separate) instruction nurses.*
Perceived workload (10) *Professionals working in the home care setting noticed a decrease in the prevalence of simple health tasks they executed due to the instructions of nursing techniques.*
Assurance of quality, uniformity and availability (11) *Professionals in the hospital indicated that the centralized approach of the API, assured the quality, uniformity and availability of instructions of nursing techniques and coaching sessions for e-health applications.*
Meaningful work (12) *Instruction nurses indicated to have experienced increased meaningfulness of their work, due to the fact that they had more time to pay personal attention to patients.*
**Level of health care systems**
Use of formal care and associated costs (13) *Patients, informal caregivers and health care professionals alike expected and/or experienced a reduction in the usage of professional care.*
Equity (14) *Professionals perceived that the activities as part of the Academy reduced the usage of professional care, enabling a more equitable division of healthcare.*
Quality of care (15) *Patients and informal caregivers experienced the activities of the API as a valuable addition or service whilst not rigorously altering their health care experience.*

#### Level of patients and informal caregivers

##### Managing illness needs: knowledge, skills and monitoring health status (thematic statement 1, 2 and 3)

All groups of participants perceived that the activities contributed to the patients’ and informal caregivers’ knowledge on their conditions and accompanying health needs, such as relevance of a health-task for their treatment, and importance of associated parameters and (alarm) symptoms. Professionals specifically pointed out paying attention to *‘hygiene*’ (H2) and *‘raising awareness’* on possible *‘complications’ (IN2)*

Additionally, participants reported that as a result of the activities, patients and informal caregivers gained specific knowledge on the execution of the skills instructed, supporting the correct completion of health tasks. To this end, patients and informal caregivers were *‘happy with the aids and the instruction’* (P4) that they were provided with, in order to help them optimally execute these health tasks.

*You need to receive an instruction, otherwise you don’t know how to do it [administer an injection]. (…) So it is nice to have such a session and to be shown and to execute it yourself.”* IC3

In line with the completion of health tasks, patients also learned to continuously monitor their health status, whether supported through an e-health application or not, and to consequently perform health promoting activities. This increased their treatment compliance. It was underscored by the instances in which patients actively deliberated whether or not to execute health tasks or to adjust their treatment regiments to manage side effects.

*Now [whilst using an application] you’re actually reminded to take that blood pressure three times a week and measure your weight, which you wouldn’t normally do. And now you do.”* P12.

*Well, what it accomplished is that a picture emerges. You get a picture of how all [parameters] move[s] over time. (…) Weight is very important to her, so that’s what you focus on the most. She can’t get above 75 kilos, so she tries to get back below that.”* IC8

##### Managing illness needs: mental well-being (thematic statement 4)

Having knowledge and skills and being able to monitor health status results in a feeling of autonomy and freedom. One patient put it as follows: *“[the instruction] leads to freedom and not to restrictions.”* (P1) These feelings leads to improved mental well-being through having ownership over ones’ health.

*You are dependent on a lot of things. And mentally that does something to you. And the moment you can take a bit of control, it improves your mental state. So in my case that’s very nice. Yes, I can really confirm that, because it seems like nothing, but it is a lot.”* P7

Additionally, patients and informal caregivers reported that being autonomous leads to less (stress in) waiting for homecare professionals and reduces the feeling of *‘the invasion of privacy’* (P7) by professionals having to come by. Patients and informal caregivers underscore the positivity of the fact that their partner or informal carer can become part of their disease management, instead of having to stand by as an outsider. However, both patients and informal caregivers do not want to burden the other too much.

*It certainly saves me stress. And I know, the homecare workers are just people too, and sometimes things come up and they are late and when they are ten minutes or fifteen minutes late I can live with that, because I’m often like that myself. But on the other hand, don’t be an hour late, because then I’m really biting my nails. I will start with putting on the stockings myself.”* P2

*Otherwise you have to wait for home care. And that’s four times a day (…) in total that will be around seventy home visits, just for a cataract surgery. Think about the costs, but also the inconvenience for yourself. I don’t know about you, but if you just sit and wait for a courier to bring a package… Just sit and wait, for four weeks, three times a day.’’* R5

##### Perceived advantages: self-efficacy, peer-to-peer contact and acknowledgement (thematic statement 5, 6 and 7)

Patients and informal caregivers reported several perceived advantages of enhanced self-management. For example, the aforementioned knowledge, completion of health tasks and execution of health promoting activities also lead to improved self-efficacy of patients and informal caregivers. Professionals hoped that partaking in the activities may lower *‘the threshold to do things independently’* (H2) in the future. Patients and informal caregivers confirmed this. They hoped that *‘they don’t need it in the future, but if so’*, they are *‘more confident’* (P7) and *‘more aware of the fact that there are always things they can do themselves’* (IC3)

Participants in the educational programs for people living with a chronic illness (Table 1, category 3), expressed their appreciation for and benefits of peer-to-peer contact.

*Above all, I saw that [the peer-to-peer contact] had a positive impact on my wife. That she could see that others could deal with it relatively casually. Because she very much has this fear of, ‘Gee, what if something happens to me’. (…) I do think it helped her find a bit of a way through that.”* IC6

Patients and informal caregivers reported that they feel acknowledged as partners in care. They report positive on the fact that a hospital recognizes that people are able to do certain activities themselves and entrust them with these activities. In turn, professionals indicated that they are *‘perhaps used to taking over things from them [patients] too often’* (IN3).

*You never think about that [doing things autonomously] if you never come to the hospital, but that they look at what you can do independently, yes, that is also a part of quality of care. I think it’s a good thing that that happens.”* IC3

##### Perceived disadvantages: complexity and burden (thematic statement 8)

Generally, the activities that are currently executed, more specifically simple nursing techniques (Table 1, category 1), are simple in the eyes of all participants. Therefore, they do not mind executing these tasks independently. Many participants expected that their willingness to independently execute a health task to decrease if they would be asked to execute a more complex skill. A patient executing a task that was classified as ‘complex’ by the API reported: *“It is simple, right? It’s just a tube with two ends: one end you attach to the bag [containing feeding], the other end you attach to the tube in your nose. It’s really not that complicated.”* (P9). This participant, however, could imagine *“when you’re anxious for everything and scared to do something wrong, it [tube feeding] might feel as quite an undertaking”* (P9). This resonated with experiences of other participants.

*“You know, it’s only a compression stocking. It’s not some sort of surgery that I have to do every day, I don’t have to change a tube or change a stoma, although you might get used to that very quickly too, but this only regards putting on a stocking.”* IC2

Carrying out health tasks independently generally takes little time, according to participants. However, sometimes even a small investment of time can have serious implications on the daily lives of participants. Additionally, participants understand they were asked to perform certain tasks, but warned that there are limits to the load that informal caregivers are willing to carry, both physically and mentally. An example of the physical load is when, for example, the health task is executed by informal caregivers who have illnesses themselves or when ergonomic considerations are not taken into account. The cognitive load could be related to scheduling or anxiousness regarding executing health tasks independently.

*You can’t say to someone: now go and put those stockings on yourself when they themselves have to get out of bed at 6.30 a.m. every day to go to work. (…) That’s when I think, in that case we have homecare professionals, they can come too. You have to have time for it, as crazy as that sounds. As I said, it’s only ten minutes, but those ten minutes can be of inconvenience.”* P2

*Oftentimes, they [the patient and the informal caregiver] do know the purpose of what we come for, with, for example, connecting and disconnecting tube feeding. They know quite well how to do it, but often they just find it too suspenseful.”* H2

*Well, I have arthrosis in my thumb and it is quite tough when putting the stockings on. But anyway, you just have to do it.”* IC2

#### Health care professionals

##### Perceived workload (thematic statement 9 and 10)

All participants expected a reduction in workload for both professionals working in the homecare provision, as well as in the hospital. The latter is in part due to the fact that ward nurses don’t have to “squeeze in” instructions themselves on busy wards, and in part due to the fact that ward nurses do not have to refer to homecare professionals anymore. In turn, transfer nurses indicated that some indications for home visits are a lot less prevalent, as opposed to earlier.

*““They [instruction nurses] relieve the workload. Because sometimes we [transfer nurses] spend a lot of time at simple home care requests, when you can’t find a suitable home care organization. We’ve experienced that so many times, that you have to call so many organizations before you find someone who can deliver that care. So I think that [the activities are] a relief, I think that’s a very clear profit for us.”* T2”

*Often those injections, that used to be something that a lot of people were referred for, to us [in home care]. That is actually a lot less already.”* H2

##### Assurance of quality, uniformity and availability (thematic statement 11)

Health care professionals within the hospital indicated that the centralized approach, in which instruction nurses have dedicated time for the activities within the API, assured the quality, uniformity and availability of activities. This is due to the fact that instruction nurses are educated in motivating and instructing and experienced in training patients and informal caregivers, have access to proper training material such as necessary *‘aids to make it [executing the health task] easier’* (IN1) and can subsequently hand out *‘information leaflets’* (IN3), and *‘have more time’* (IN1) to educate patients and informal caregivers.

*They take over a part of our work, and I really like that. It also just makes me feel nicer for the patients. That I know, okay, they can now have their attention, get a calm explanation, they can ask questions straight away. Yes, that just feels nicer than when I’m s"tanding there and my pager goes off ten times and then I have to leave and return again.”* R10

As for health professionals working in home care: they have indicated that the training does not affect them directly and hence find it difficult to judge the impact thereof on their working conditions. Home care nurses suggested that education is an integral part of their work, and underscore the importance of *‘working together’* (H1) in transitional care.

*I don’t see how many referrals should have come, as compared to how many [patients] actually were referred to us.”* R11

##### Meaningful work (thematic statement 12)

According to patients and informal caregivers, the independent execution of health tasks leads to an improvement of meaningfulness of work for healthcare professionals. This resonates with the experiences of instruction nurses, who indicated to have more time to pay personal attention to patients as compared to ward nurses and now can optimally support patients and informal caregivers in self-management.

*And the additional attention I can now give [as an instruction nurse] at the Academy. (…) And the patients love it, and they ask me: will you come back tomorrow? (…) It’s not just having fun, it’s more the attention that the patient just misses, because the nursing staff doesn’t have time for it.”* IN4

#### Health care systems

##### Use of formal care and associated costs (thematic statement 13)

Patients, informal caregivers and health care professionals alike expected or experienced a reduction in the usage of professional care. This could be either in terms of *‘shortened hospital stays’* (TN2), *‘less visits’* (H1) by home care or a *‘reduction of out-patient hospital visits’* (IN2). The use of e-health applications has the potential of preventing *‘hospitalizations of patients’* (R9). However, this type of care is currently executed on-top of normal care as it is not yet embedded in the care path, possibly increasing costs rather than a reducing them.

*It has helped, that sometimes people go home without home care. And sometimes with significantly reduced home care. And that’s just nice because, well it’s fairly known, that there’s shortage in health care. And that’s very evident in home care.”* TN2

*Top-on means: people get regular care through out-patient visits, and the SanaCoach comes as an extra. It does not replace out-patient visits.”* R2

##### Equity (thematic statement 14)

Participants indicated that as a result of patients and informal caregivers independently executing tasks and thus not needing help from professionals, healthcare professionals are able to redirect their attention to people who need it most. This can be more complex cases or people without informal caregivers who can take care for them. This was substantiated by transfer nurses: they see an increase in *´patients with more problems´* (TN2) and when a part of their population can independently execute health tasks, they are given *´more space to devote their time to complex cases´* (TN2). Lastly, professionals also warned for *‘the loneliness of elderly’* (IN1), as for some people the visit of the home care nurse is the only social contact they might have in a day.

*There will be more time for the individual behind some doors who needs more extensive home care. The person with dementia that is still living at home independently. (…) It is nice to have space to drink a cup of coffee with these people, because now you do not need to go and change a stoma or change tube feeding, as those people can now do it independently.”* R11

##### Quality of care (thematic statement 15)

In terms of care experience, patients and informal caregivers indicated to experience the activities of the API as a *‘nice addition’* (P5), but overall the activities did not rigorously alter their health care experience. Health care professionals within the hospital characterized the activities as a *´service’* (R2) to patients and informal caregivers.

## Discussion

Our study explored the experiences of participating patients, their informal caregivers and health care professionals – in and outside of the hospital – with the Academy for Patients and Informal caregivers as a comprehensive infrastructure to promote self-management through active patient involvement in treatment, e-health and self-management programs. In the following sections we will discuss the results in relation to the two empirical concepts that formed the basis of our semi-structured interview guide, self-management and Quadruple aim. Subsequently, we discuss our findings in the light of patient- and family centered care and the participative role of informal caregivers and associated burden.

Patients and informal caregivers indicated that they felt enabled to manage illness needs independently, including correctly completing (simple) health tasks and monitoring their health status. Patients and informal caregivers experienced a subsequent increase in mental wellbeing and self-efficacy, and felt acknowledged as a partner in care. Schulman-Green and colleagues formulated three different categories of self-management processes with accompanying tasks and skills [13]. Many of the skills and tasks required for the first category of self-management processes, focusing on illness needs, such as learning skills, monitoring symptoms, developing confidence and self-efficacy, correspond with the experiences described by participants in this study. Furthermore, these findings resonate with the ‘improving clinical outcomes’ aim as part of the Quadruple aim [24].

The second self-management process, activating resources, such as healthcare resources using e-health applications and social resources in terms of peer-support, were also reported by participants in this study. To a lesser extent, skills and tasks as part of the process of living with a chronic illness were reported, partly due to the fact that most of the patients and informal caregivers who were interviewed had diseases of limited duration, as opposed to chronic diseases with more consistent symptoms. The instruction nurses working at the API experienced an increase in the meaningfulness of their work, resonating with the ‘improving work life of clinicians’ part of the Quadruple aim. On health system level, participants in this study reported a decrease in use of formal healthcare whilst obtaining a more equitable division of care and maintaining an equal, generally positive, health care experience. The latter findings resonate with the last two aims of the Quadruple aim, namely reducing costs and improving patient experience. Additionally, the principle of equity was touched upon by the participants and unearthed as a theme in data analysis. This extended the findings from the Quadruple Aim, to the further expanded Quintuple Aim [29]. Overall, the qualitative findings in this study echo with the five aims as part of the Quintuple Aim, that forms an updates compass to optimize health system performance originating from the Quadruple Aim.

The independent execution of health tasks and subsequent self-management is an acceptable burden for the participants in this study. Participants in our study anticipated an increasing burden, the more complex health tasks become. Recently, several initiatives have been developed in which essential nursing care activities are taught to informal caregivers, in for example surgical wards and intensive care units [30, 31]. They report that active participation in care by informal caregivers was experienced as an acceptable burden, but holds the danger of losing touch with oneself for informal caregivers [32, 33]. Our study adds to these findings with experiences from a transitional care setting as opposed to a clinical setting. Furthermore, our study describes experiences with nursing techniques, such as the described administration of subcutaneous injections or putting on stockings, as opposed to fundamental care activities such as support with personal cleansing and support with eating and drinking. However, caregiver burden is expected to become increasingly prevalent in the light of an aging population and the lack of formal support for informal caregivers. Especially spousal caregivers face greater challenges as compared to adult children assisting a parent, due to the fact that they, in almost all cases, live with the care recipient, feel that they have little to no choice other than to take up the care responsibilities and are more vulnerable as they are older themselves [34]. Additional training for instruction nurses in self-management support, specifically focusing on (the determinants and risk factors of) caregiver burden seem paramount in light of these demographic changes. Additionally, self-management programs for informal caregivers could be deployed [35].

### Strengths and limitations

One of the main strengths of the presented study is the incorporation of the views of patients, informal caregivers and health care professionals to triangulate the results. Additionally, data were iteratively analyzed and discussed within the research team, providing the researchers with the opportunity to validate the findings. Moreover, data were collected over a long period of time (i.e. 18 months), leading to a more comprehensive and nuanced understanding, as well as adding to the consistency of our findings. Furthermore, participants actively participated in or referred to the entire set of activities executed as part of the API, providing a comprehensive description of experiences. However, a limitation of this study might be that not each activity has been as thoroughly explored, which implies that data sufficiency might not have been obtained for each activity within the API. For example, only two participants of educational programs for living with a chronic illness participated. In our analysis we focused on all processes as parts of self-management and the findings for different categories seem consistent with these processes. Therefore, we think that the omission of themes due to the fact that data sufficiency might not be reached for each individual activity, is limited. Also, our sample of participants was relatively well-educated and young, came from one hospital and had a Dutch, cultural background. Therefore, our sample was homogeneous and different cultural backgrounds were not explored. This seems relevant, as age, cognitive abilities and cultural beliefs have been identified as factors affecting self-management [14]. It remains unknown whether older, frailer or more culturally diverse groups have similar experiences with this intervention as the current group did. We hypothesize that execution of the activities in an older, frailer or culturally diverse population might be less feasible in its current form. However, through the training of younger, well-educated participants, a larger share of formal care remains available to others – potentially needing it more – making healthcare more equitable. Exploring the motives of patients and informal caregivers who declined partaking in an activity or the motives of physicians not to ask specific groups to partake, might offer valuable additional insights to which population this intervention is applicable to.

## Conclusion

The Academy for Patients and Informal caregivers has the potential to adequately support patients and informal caregivers’ self-management, whilst also contributing to meaningfulness of work of healthcare professionals, reducing the appeal to formal care and possibly advancing equity in healthcare. The burden of self-management seems acceptable for participants, but the growing burden on informal caregivers should be taken into consideration in the future direction of development of the Academy of Patients and Informal caregivers. This can be operationalized through the training of instruction nurses on this topic and involve the informal caregiver in the shared decision making process when deliberating to participate in one of the activities of the Academy for Patients and Informal caregivers.

### Electronic supplementary material

Below is the link to the electronic supplementary material.

Supplementary Material 1

## Data Availability

A copy of the code tree is provided in the Additional file 3. Anonymized transcripts are available in Dutch upon well-founded request.

## Footnotes

1 Instruction nurses work at the API and have had additional training in didactic skills, motivational interviewing and specific train-the-training courses for educational programmes to optimally equip them to support patients and informal caregivers´ self-management.
