# Supplementary material for: Patients and informal caregivers in the lead: a qualitative study on the experiences of patients, informal caregivers, and healthcare professionals with involvement in treatment, e-health and self-management programs
Source: BMC Health Serv Res. 2024 Jun 10;24:713. doi: 10.1186/s12913-024-11156-z (PMC11165740; doi:10.1186/s12913-024-11156-z)
Supplement: Supplementary file 1 — Supplementary Material 1 [file 12913_2024_11156_MOESM1_ESM.docx]

**Additional file 1 – Semi-structured interview guide for patients and informal caregivers
Opening:**

- For this study, we are interested in what influence the instruction of the Academy for Patients and Informal caregivers has on your ability to take charge of your own health. Self-management is a broad concept: so what do you understand by self-management?

**Key questions:**

- What did the instruction benefit and/or cost you and/or your informal carer? Did the instruction help you to take control of your own health/as an informal caregiver? In what way (not)?
  - In your experience, what is the influence of the activities of the Academy for Patients and Informal caregivers on the categories of self-management processes:
    - Focusing on illness needs;
    - Activating resources;
    - Living with a chronic illness?
- What influence has the instruction had on how you feel about your health? Is that influence positive or negative? Why?
  - If applicable: Has the instruction also influenced the health of your informal caregiver?
- What influence has the instruction had on your perceived quality of care? Have you started to appreciate the care better or worse? Why?
  - Did you feel safe to execute the tasks yourself? Why (not)?
  - If applicable: Has the instruction had any influence on the perceived quality of care by your informal caregiver?
- What influence do you think the instruction has had on the job satisfaction of the professionals? Why?
- What influence has the instruction had on the use of care? Has it increased or decreased? Why and how? Do you expect this to be different in the future?
  - If applicable: What influence has the instruction had on your informal caregiver?

**Additional and closing questions:**

- Are there any issues you would like to discuss that have not yet been discussed?

Thank you for your participation. You will be sent a summary of this session with the invitation to make adjustments or additions.

**Additional file 2 – Semi-structured interview guide for health care professionals**

**Opening:**

- For this study, we are interested in what influence the instruction of the Academy for Patient and Informal caregivers has on your ability to take charge of your own health. Self-management is a broad concept: so what do you understand by self-management? And how can one enhance self-management?

**Key questions:**

- What are your own experiences with the Academy for Patients and Informal caregivers?
- In your opinion, what do patients and/or informal caregivers achieve regarding self-management after participating in one of the activities as part of the Academy for Patients and Informal caregivers?
  - In your experience, what is the influence of the activities of the Academy for Patients and Informal caregivers on the categories of self-management processes:
    - Focusing on illness needs;
    - Activating resources;
    - Living with a chronic illness?
- What, in your experience, is the impact of the academy's patient and caregiver trainings on the participant's health?
- What impact, in your opinion, do the activities of the Academy for Patients and Informal caregivers have on the patient’s and/or informal caregivers’ perceived quality of care?
- What is, in your opinion, the impact of the discussed activities on the job satisfaction of the healthcare professionals?
- What is, in your opinion, the impact of the discussed activities on the use of care and associated health care costs?

**Additional and closing questions:**

- Are there any issues you would like to discuss that have not yet been discussed?

Thank you for your participation. You will be sent a summary of this session with the invitation to make adjustments or additions.

**Additional file 3 – coding tree**

1. Healthcare
   1. Quality of care
   2. Costs
   3. Extent of care use
      1. Patient flow within the hospital
      2. Less or shorter use of home care
      3. Less of shorter hospital visits
   4. Work force shortage
2. Informal caregivers
   1. Burden
      1. Complexity of the health task
   2. Influence on health
   3. Insight into possibilities
   4. Knowledge and understanding
   5. Feeling safe and/or anxious
      1. Anxiety of hurting the other person
   6. Independence
3. Patients
   1. Burden
      1. Complexity of the health task
   2. Involvement of informal caregiver
   3. Influence on health
   4. Insight into possibilities
   5. Knowledge and understanding
   6. Quality and safety
   7. Peer-to-peer contact
   8. Personal attention
   9. Personal growth
   10. Feeling safe and/or anxious
       1. Anxiety of hurting the other person
   11. Independence
       1. Ownership
4. Healthcare professionals
   1. Workload and job satisfaction
      1. Meaningful work and gratitude
      2. Physical impact
   2. Quality of activities
   3. Confidence in independence
